# Analysis of the correlation between the longitudinal trajectory of SOFA scores and prognosis in patients with sepsis at 72 hour after admission based on group trajectory modeling

**DOI:** 10.1016/j.jointm.2021.11.001

**Published:** 2021-12-21

**Authors:** Rui Yang, Didi Han, Luming Zhang, Tao Huang, Fengshuo Xu, Shuai Zheng, Haiyan Yin, Jun Lyu

**Affiliations:** 1 Department of Intensive Care Unit, The First Affiliated Hospital of Jinan University, Guangzhou, Guangdong 510630, China; 2 School of Public Health, Xi'an Jiaotong University Health Science Center, Xi'an, Shaanxi 710061, China; 3 School of Public Health, Shaanxi University of Chinese Medicine, Xianyang, Shaanxi 712046, China; 4 Department of Clinical Research, The First Affiliated Hospital of Jinan University, Guangzhou, Guangdong 510630, China

**Keywords:** Sepsis, Sequential organ failure assessment score, Group-based trajectory model, Medical information mart for intensive Care (MIMIC)-IV database, Survival analysis

## Abstract

**Background:**

To identify the distinct trajectories of the Sequential Organ Failure Assessment (SOFA) scores at 72 h for patients with sepsis in the Medical Information Mart for Intensive Care (MIMIC)-IV database and determine their effects on mortality and adverse clinical outcomes.

**Methods:**

A retrospective cohort study was carried out involving patients with sepsis from the MIMIC-IV database. Group-based trajectory modeling (GBTM) was used to identify the distinct trajectory groups for the SOFA scores in patients with sepsis in the intensive care unit (ICU). The Cox proportional hazards regression model was used to investigate the relationship between the longitudinal change trajectory of the SOFA score and mortality and adverse clinical outcomes.

**Results:**

A total of 16,743 patients with sepsis were included in the cohort. The median survival age was 66 years (interquartile range: 54–76 years). The 7-day and 28-day in-hospital mortality were 6.0% and 17.6%, respectively. Five different trajectories of SOFA scores according to the model fitting standard were determined: group 1 (32.8%), group 2 (30.0%), group 3 (17.6%), group 4 (14.0%) and group 5 (5.7%). Univariate and multivariate Cox regression analyses showed that, for different clinical outcomes, trajectory group 1 was used as the reference, while trajectory groups 2–5 were all risk factors associated with the outcome (*P* < 0.001). Subgroup analysis revealed an interaction between the two covariates of age and mechanical ventilation and the different trajectory groups of patients’ SOFA scores (*P* < 0.05).

**Conclusion:**

This approach may help identify various groups of patients with sepsis, who may be at different levels of risk for adverse health outcomes, and provide subgroups with clinical importance.

## Introduction

Sepsis is particularly common in the intensive care unit (ICU) and is a major cause of disability and death in critically ill patients. Studies have shown that the mortality of patients with sepsis in the ICU is 10–40%.[1], [2] Sepsis affects at least 3 million people worldwide each year. Severe sepsis refers to patients with sepsis associated with organ dysfunction, hypotension, poor tissue perfusion and other conditions.

In 2016, the Sequential Organ Failure Assessment (SOFA) score was listed as the diagnostic criterion for sepsis (Sepsis 3.0).^[3]^ As the most commonly used critical disease scoring system in clinical practice, numerous large retrospective studies have demonstrated that the SOFA scoring system is effective in evaluating the prognosis of patients with sepsis.[4], [5], [6]

Previous studies have also shown that patient age, SOFA score, and APACHE score are independent risk factors for prognosis.[7], [8], [9] It has also been demonstrated that change in the difference between the SOFA scores at two time points predicts the 28-day prognosis of patients with sepsis.^[10]^ Previous studies utilizing latent growth mixture modeling have investigated the impact of changes in SOFA score on the development of sepsis into persistent critical illness. The present study was based on a large critical illness database to achieve population clustering through the different laws of the longitudinal development of SOFA. This analysis was aimed at a more accurate identification of patients with sepsis at high risk of experiencing adverse outcomes. Longitudinal change trajectory is used to describe the process of continuous and dynamic changes in the development of a certain object over time. This trajectory has been widely used in medicine, psychology, and other related fields.^[11]^ In 1999, Nagin.^[12]^ supported the use of group-based trajectory modeling (GBTM) for the analysis of longitudinal data. As a special application of the finite mixed model, this method is used to analyze the similarity of certain behaviors or results over time or age, and subsequently divide individual subjects into groups.^[13]^ This model is used to explore the presence of heterogeneity in the whole population.

In this study, patients with sepsis in the Medical Information Mart for Intensive Care (MIMIC)-IV database, who had a record of SOFA scores at 72 h, were included as the research population. The trajectory model from GBTM was used to construct a traditional univariate trajectory model for the 72-h SOFA score. The Cox proportional hazard regression model was used to explore the relationship between each trajectory and the risk of adverse outcomes. This study aimed to determine the relationship between SOFA score trajectory and poor prognosis in patients with sepsis in the ICU.

## Methods

### **Data source and selection of participants**

The MIMIC-IV database^[14]^ is an updated version of MIMIC-III. This database currently contains comprehensive high-quality data of ICU patients at the Beth Israel Deaconess Medical Center from 2008 to 2019.[15], [16] According to the Sepsis-3 criteria, sepsis is a suspected infection combined with an acute increase in the SOFA score ≥2.^[3]^ According to the diagnostic criteria, we included patients aged >18 years, with ICU stay >24 h, and with complete SOFA score records per hour for the first 72 h after admission.

### **Variables and endpoints**

The following basic information of the patients with sepsis were extracted: age, sex, race, marital status, insurance status, use of mechanical ventilation, use of renal replacement therapy (RRT), administration of glucocorticoids, and the Charlson Comorbidity Index (CCI). Vital signs included the heart rate, respiratory rate, systolic blood pressure (SBP), diastolic blood pressure (DBP), mean arterial pressure (MBP), body temperature, and pulse oxygen saturation (SPO_2_). The first laboratory test parameters of patients with sepsis collected within 24 h after admission to the ICU were partial pressure of carbon dioxide (PaCO_2_), partial pressure of oxygen (PaO_2_), white blood cell count (WBC), bicarbonate, chloride, hematocrit, blood urea nitrogen and creatinine ratio (BCR), international normalized ratio (INR), red cell distribution width (RDW), anion gap, hemoglobin, and platelets. Data on the length of stay in the hospital and ICU were also extracted.

The primary endpoint was the survival status on days 7 and 28 in the hospital and ICU. The secondary endpoint was the occurrence of adverse prognostic events, such as septic shock and acute respiratory failure (ARF).

### **GBTM**

GBTM is mainly used to track the data analysis of a heterogeneous population. The principle of this process is to explore the subgroups with different development trajectories in the population, and subsequently analyze the developmental trend of different subgroups. The level and shape of the trajectory are determined by the regression parameters of the model, which are estimated by the maximum likelihood ratio.[17], [18] GBTM can adapt to various data distributions, such as binary logistic regression, Poisson, zero-inflated Poisson, and normal distribution.^[19]^

In our study, the fitting basis of SOFA trajectory grouping based on GBTM model is mainly determined by the following indicators:^[20]^ (1) Bayesian information criterion (BIC), e.g., BIC values closer to 0 indicate a better fitting effect. (2) Average post-test grouping probability (Avepp), in which the index reflects the post-test probability of each individual being classified into the corresponding trajectory subgroup, with >0.7 typically used as the acceptance criterion. (3) Bayesian factor logarithmic value (2log _e_[B10]), which is approximately equal to twice the difference between the BIC values (2ΔBIC) of the two comparison models. A value >6 indicates that the fitting results of the two models are different, which is acceptable. For more complex models, if the value is <2, a more concise model can be accepted. (4) ≥10% of patients are classified in each trajectory. (5) Appropriate correspondence between the estimated probability of the group and the proportion of assigned members. The simplicity and clinical rationality of the model are also considered.

### **Statistical analysis**

The PostgreSQL 10.7,^[21]^ Navicat Premium 11.0 software,^[22]^ and Structured Query Language (SQL)^[23]^ were used to extract the above observation indicators from the MIMIC-IV database. Continuous data are presented as the mean ± standard deviation or median (interquartile). Categorical variables were described as percentages and compared using the *χ*^2^ test. Comparisons between the two groups were performed using Student's *t*-test (normal distribution) or the Mann–Whitney *U* test (non-normal distribution). Cumulative incidence function curves of different outcomes were plotted and compared using the log-rank test. The Cox proportional hazards regression model was used to determine significant differences in terms of survival status and adverse events following stratification of patients according to their different SOFA score trajectories.

Three multivariate models were used to evaluate the prognostic value of the SOFA score trajectory for each study endpoint. In model I, covariates were only adjusted for age, sex, ethnicity, insurance, and marital status. In model II, covariates of vital signs were only adjusted for model I plus SBP, DBP, MBP, respiratory rate, temperature, SPO_2_, PaCO_2_, and PaO_2_. In model III, covariates of laboratory parameters were adjusted for model II plus glucose, RRT, mechanical ventilation, administration of glucocorticoids, CCI, WBC, bicarbonate, chloride, hematocrit, BCR, INR, RDW, anion gap, hemoglobin, and platelets. The results were expressed as the hazard ratio (HR) and its 95% confidence interval (CI).

All statistical analyses were performed with R software (version 4.0.5, Lucent Technologies, USA) and Stata (version 16.1, Stata Corp, College Station, TX, USA). *P*-values <0.05 (two-sided) denoted statistically significant differences.

## Results

### **Baseline demographic data, clinical data, and outcomes**

A total of 16,743 patients with sepsis were included in the study. The characteristics of the clinical groups are shown in detail in Table 1. Based on the comprehensive evaluation of model fitting effect, the trajectory grouping for the GBTM analysis of changes in the 72-h SOFA scores in patients with sepsis yielded five groups: group 1 (*n* = 5503, 32.8%), group 2 (*n* = 5004, 30.0%), group 3 (*n* = 2947, 17.6%), group 4 (*n* = 2342, 14.0%) and group 5 (*n* = 947, 5.7%). Patients in group 1 exhibited the lowest SOFA score, showing a trend of initial increase, followed by reduction and subsequent increase within 72 h. Patients in group 2 had a higher SOFA score than those in group 1, showing a fluctuating trend of initial increase and subsequent stabilization. Patients in group 3 had SOFA scores ranging between those recorded in groups 1 and 2, showing an initial increase, followed by a sharp decline within 72 h, and finally continuing to rise. Patients in groups 4 and 5 exhibited relatively high SOFA scores, with similar trends of change.

**Table 1:** Fit statistics for different number of trajectory groups.

Trajectory groups	Best BIC	Best AIC	2ΔBIC	Likelihood	AvePP (per trajectory group)	Percent of patients with posterior probability <70% (per trajectory group)	Percent of sample size (per trajectory group)	Relative entropy
1	−443,433.2	−443,413.9	NA	−443,408.9	1	0	100	NA
2	−393,822.8	−393,784.2	99,220.9	−393,774.2	0.99/0.98	0.3/0.7	69/31	0.951
3	−373,887.9	−373,830.0	39,869.7	−373,815.0	0.97/0.96/0.98	0.5/0.6/0.9	49/37/14	0.924
4	−365,471.5	−365,394.2	16,832.8	−365,374.2	0.96/0.94/0.95/0.97	0.6/0.7/0.8/0.9	37/35/20/8	0.916
5	−360,994.9	−360,898.4	8953.2	−360,873.4	0.95/0.91/0.91/0.95/0.97	0.7/0.7/0.8/0.9/0.9	33/30/18/14/6	0.884
6	−356,096.4	−355,980.5	9797.0	−355,950.5	0.94/0.89/0.90/0.92/0.95/0.97	0.7/0.9/0.8/0.9/0.9/0.9	28/12/28/16/12/4	0.897
7	−353,099.9	−352,964.7	5993.0	−352,929.7	0.94/0.86/0.90/0.88/0.91/0.95/0.98	0.8/0.8/0.9/0.9/0.9/0.9/1.0	24/20/12/18/12/10/4	0.886

AIC: Akaike Information Criterion; AvePP: Average post-test grouping probability; BIC: Bayesian information criterion; NA: Not avalible; 2ΔBIC: Twice the difference between the BIC values.

The median survival age was 66 years (interquartile range: 54–76 years). The population included 9575 men (57.2%) and 7168 women (42.8%). The majority of patients was White (65.9%) and married (42.9%), and had received mechanical ventilation therapy (95.9%). The in-hospital mortality rates on days 7 and 28 were 6.0% (*n* = 934) and 17.6% (*n* = 2962), respectively. The mortality rates among patients in the ICU were 8.1% (*n* = 1372) and 19.1% (*n* = 3202), respectively. The incidence rates of septic shock and ARF were 11.5% (*n* = 1932) and 42.8% (*n* = 7178), respectively.

### **GBTM of the SOFA score**

**Figure 1:**
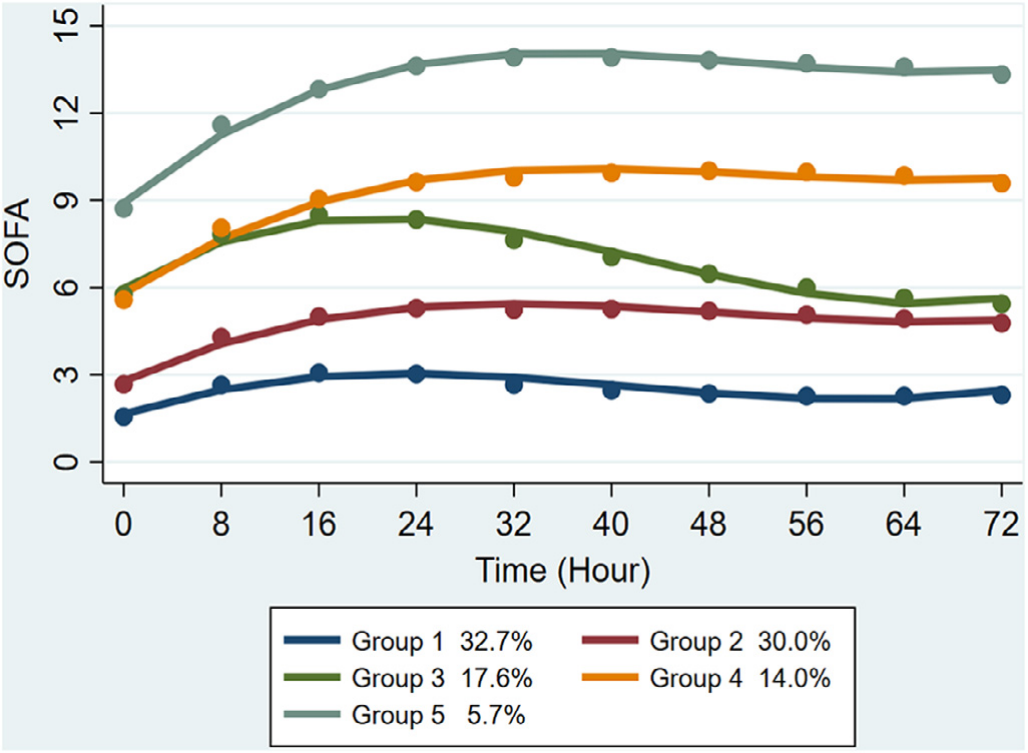
Five trajectories of the SOFA score based on GBTM. GBTM: Group-based trajectory modeling; SOFA: Sequential Organ Failure Assessment.

**Figure 2:**
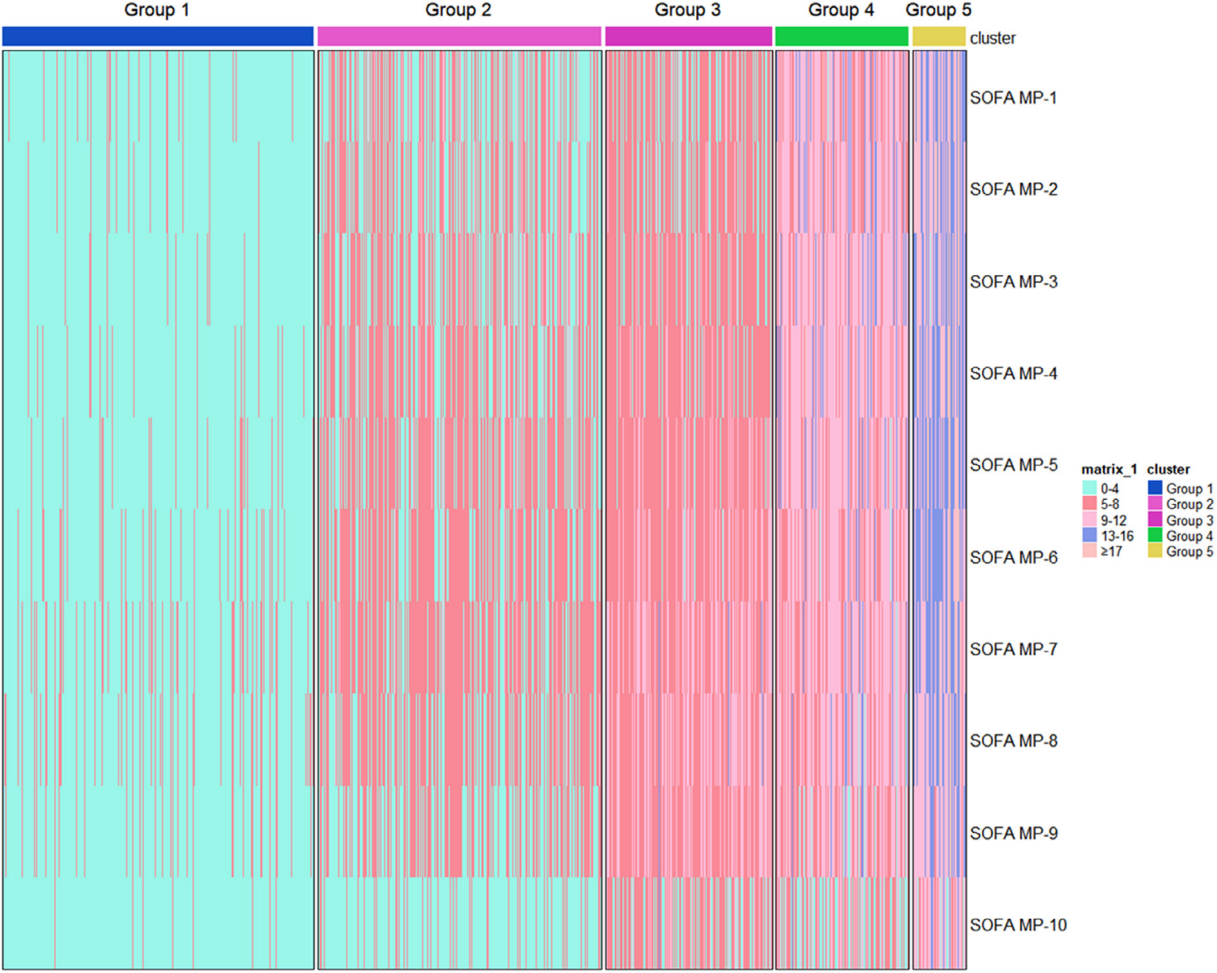
Heatmap of the total SOFA score over time for the five identified groups. Each patient is represented by a single line colored according to the legend, depicting SOFA score intervals, complications, or death in the hospital. SOFA score group (*y*-axis); trajectory group (*x*-axis). SOFA-MP: SOFA-measurement period; SOFA: Sequential Organ Failure Assessment.

A univariate censored normal (CNORM) trajectory model was fitted according to the 72-h SOFA score level of the longitudinal cohort population. The SOFA scores of patients in groups 1–5 were gradually increased, with overlapping SOFA trajectories for groups 2 and 3 at the time of admission [Supplementary Table S1 and Supplementary Fig. S1]. The 72-h SOFA score trajectories of the five groups of patients are shown in Figure 1. In addition, the heatmaps of the SOFA score trajectories in the five groups were plotted [Figure 2]. The coefficients of cubic functions in the five groups are shown in Supplementary Table S1.

The baseline characteristics of patients stratified based on the five trajectory groups are shown in Table 2. Compared with group 1, higher SOFA scores of patients in the trajectory groups 2–5 were linked to a greater proportion of males and more frequent use of RRT and mechanical ventilation. Overall, there were statistically significant differences in the baseline characteristics of patients across the five trajectory groups (*P* < 0.05).

**Table 2:** Descriptive characteristics of overall participants and by SOFA trajectory.

Variables	Total (*n* = 16,743)	SOFA score					*F/*χ^2^	*P-*value
		Group 1 (*n* = 5503)	Group 2 (*n* = 5004)	Group 3 (*n* = 2947)	Group 4 (*n* = 2342)	Group 5 (*n* = 947)		
Age (years)	66 (54.76)	65 (53.76)	68 (56.78)	67 (56,.77)	64 (54.75)	58 (49.68)	61.7	<0.010
Sex							65.4	<0.001
Male	9575 (57.2)	2934 (53.3)	2859 (57.1)	1771 (60.1)	1423 (60.8)	588 (62.1)		
Female	7168 (42.8)	2569 (46.7)	2145 (42.9)	1176 (39.9)	919 (39.2)	359 (37.9)		
Ethnicity							55.4	<0.001
White	11,037 (65.9)	3690 (67.1)	3333 (66.6)	1964 (66.6)	1495 (63.8)	555 (58.6)		
Black	1704 (10.2)	524 (9.5)	514 (10.3)	325 (11.0)	258 (11.0)	83 (8.8)		
Other	402 (23.9)	1289 (23.4)	1157 (23.1)	658 (22.3)	589 (25.1)	309 (32.6)		
Insurance							145.7	<0.001
Medicaid	1235 (7.4)	437 (7.9)	302 (6.0)	209 (7.1)	181 (7.7)	106 (11.2)		
Medicare	8156 (48.7)	2498 (45.4)	2591 (51.8)	1555 (52.8)	1177 (50.3)	335 (35.4)		
Other	7352 (43.9)	2568 (46.7)	2111 (42.2)	1183 (40.1)	984 (42.0)	506 (53.4)		
Marital status							19.7	0.011
Married	7188 (42.9)	2332 (42.4)	2157 (43.1)	1279 (43.4)	1041 (44.4)	379 (40.0)		
Single	4712 (28.1)	1591 (28.9)	1337 (26.7)	814 (27.6)	667 (28.5)	303 (32.0)		
Other	4843 (28.9)	1580 (28.7)	1510 (30.2)	854 (29.0)	634 (27.1)	265 (28.0)		
RRT							3623.6	<0.001
Yes	2662 (15.9)	104 (1.9)	493 (9.9)	555 (18.8)	875 (37.4)	635 (67.1)		
No	14,081 (84.1)	5399 (98.1)	4511 (90.1)	2392 (81.2)	1467 (62.6)	312 (32.9)		
Mechanical ventilation							156.8	<0.001
Yes	16,054 (95.9)	5144 (93.5)	4807 (96.1)	2858 (97.0)	2303 (98.3)	942 (99.5)		
No	689 (4.1)	359 (6.5)	197 (3.9)	89 (3.0)	39 (1.7)	5 (0.5)		
Corticosteroids							48.6	<0.001
Yes	3945 (23.6)	1451 (26.4)	1164 (23.3)	587 (19.9)	519 (22.2)	224 (23.7)		
No	12,798 (76.4)	4052 (73.6)	3840 (76.7)	2360 (80.1)	1823 (77.8)	723 (76.3)		
CCI	6.0 (4.0, 8.0)	5.0 (4.0, 7.0)	6.0 (4.0, 8.0)	7.0 (5.0, 9.0)	7.0 (5.0, 9.0)	6.0 (5.0, 8.0)	4694.4	<0.001
Glucose (mg/dL)	132.0 (106.0, 172.0)	127.0 (105.0, 161.0)	132.0 (107.0, 171.0)	136.0 (109.0, 182.0)	138.0 (107.0, 185.0)	132.0 (99.0, 192.0)	24.4	<0.001
Heart rate (beats/min)	91.0 (78.0, 107.0)	90.0 (77.0, 106.0)	90.0 (77.0, 105.0)	90.0 (79.0, 105.0)	94.0 (80.0, 109.0)	98.0 (82.0, 113.50)	27.7	<0.001
SBP (mmHg)	112.0 (104.0, 124.0)	119.0 (109.0, 131.0)	113.0 (105.0, 126.0)	109.0 (103.0, 117.0)	107.0 (101.0, 116.0)	104.0 (99.0, 112.0)	407.8	<0.001
DBP (mmHg)	60.0 (54.0, 67.0)	64.0 (57.0, 71.0)	60.0 (54.0, 67.0)	58.0 (52.0, 64.0)	59.0 (53.0, 65.0)	57.0 (51.0, 63.0)	267.7	<0.001
MBP (mmHg)	75.0 (69.0, 82.0)	79.0 (72.0, 87.0)	75.0 (70.0, 82.0)	73.0 (68.0, 78.0)	73.0 (68.0, 78.0)	71.0 (66.0, 76.0)	325.8	<0.001
Respiratory rate (beats/min)	19.0 (16.0, 24.0)	19.0 (16.0, 24.0)	19.0 (16.0, 24.0)	19.0 (15.0, 24.0)	20.0 (16.0, 24.0)	20.0 (16.0, 25.0)	436.9	<0.001
SpO_2_	98.0 (95.0, 10.0)	98.0 (95.0, 10.0)	98.0 (95.0, 10.0)	98.0 (95.0, 10.0)	98.0 (94.0, 10.0)	97.0 (94.0,10.0)	0.8	<0.001
Temperature ( °C)	98.3 (97.6, 99.0)	98.3 (97.7, 99.0)	98.2 (97.6, 98.9)	98.3 (97.6, 99.0)	98.2 (97.6, 98.9)	98.1 (97.5, 98.8)	11.8	<0.001
PaCO_2_ (mmHg)	41.0 (35.0, 48.0)	41.0 (35.0, 48.0)	41.0 (35.0, 48.25)	42.0 (36.0, 50.0)	41.0 (34.0, 49.0)	40.0 (33.0, 48.0)	16.7	<0.001
PaO_2_ (mmHg)	96.0 (59.0, 174.0)	104.0 (64.0, 187.0)	95.0 (59.0, 175.0)	97.0 (57.0, 175.0)	88.0 (53.0, 158.0)	85.0 (53.5, 133.0)	22.6	<0.001
WBC (10^9^/L)	12.0 (8.3, 16.9)	11.6 (8.5, 15.5)	11.7 (8.2, 16.4)	13.1 (8.4, 18.8)	12.5 (8.0, 18.4)	12.2 (7.4, 19.1)	24.4	<0.001
Bicarbonate (mg/dL)	23.0 (20.0, 26.0)	24.0 (21.0, 27.0)	23.0 (20.0, 26.0)	22.0 (19.0, 25.0)	20.0 (17.0, 24.0)	19.0 (15.0, 22.0)	453.4	<0.001
Chloride (mEq/L)	104.0 (99.0, 108.0)	104.0 (10.0, 108.0)	104.0 (10.0, 109.0)	105.0 (10.0, 110.0)	104.0 (99.0, 109.0)	102.0 (97.0, 107.0)	25.9	<0.001
Hematocrit	31.3 (27.1, 36.0)	32.5 (28.5, 36.8)	31.1 (27.0, 35.8)	30.7 (26.9, 35.4)	30.2 (25.8, 35.5)	28.4 (24.4, 34.6)	65.3	<0.001
BCR	20.0 (14.5, 27.3)	20.0 (15.5, 27.5)	20.8 (15.0, 27.8)	20.0 (14.1, 26.9)	18.8 (13.0, 26.3)	16.3 (11.2, 23.5)	27.5	<0.001
INR	1.3 (1.1, 1.6)	1.2 (1.1, 1.4)	1.3 (1.1, 1.5)	1.3 (1.2, 1.6)	1.5 (1.2, 1.9)	1.8 (1.5, 2.5)	181.3	<0.001
RDW	15.1 (13.8, 16.9)	14.4 (13.4, 16.0)	15.1 (13.9, 16.8)	15.4 (14.2, 17.2)	15.9 (14.5, 17.9)	16.5 (14.8, 19.1)	304.7	<0.001
Anion gap	15.0 (12.0, 18.0)	14.0 (12.0, 16.0)	14.0 (12.0, 17.0)	15.0 (12.0, 18.0)	17.0 (14.0, 20.0)	19.0 (16.0, 24.0)	555.7	<0.001
Hemoglobin (g/dL)	10.2 (8.8, 11.9)	10.7 (9.2, 12.2)	10.2 (8.7, 11.8)	10.0 (8.7, 11.5)	9.8 (8.4, 11.4)	9.4 (8.0, 11.4)	76.0	<0.001
Platelet (10^9^/L)	190.0 (130.0, 264.0)	223.0 (172.0, 297.0)	191.0 (137.0, 262.0)	170.0 (115.0, 249.0)	147.0 (90.0, 221.0)	98.0 (58.5, 151.5)	380.1	<0.001

Continuous data are presented as median (interquartile range), whereas categorical data are presented as *n* (%).

BCR: Blood urea nitrogen and creatinine ratio; CCI: Charlson Comorbidity Index; DBP: Diastolic blood pressure; INR: International normalized ratio; MBP: Mean arterial pressure; PaCO_2_: Artial pressure of carbon dioxide; RRT: Renal replacement therapy; RDW: Red cell distribution width; SPO_2_: Oxygen saturation; SBP: Systolic blood pressure; SOFA: Sequential Organ Failure Assessment; WBC: White cell count.

### **Survival analysis in the trajectory groups**

**Figure 3:**
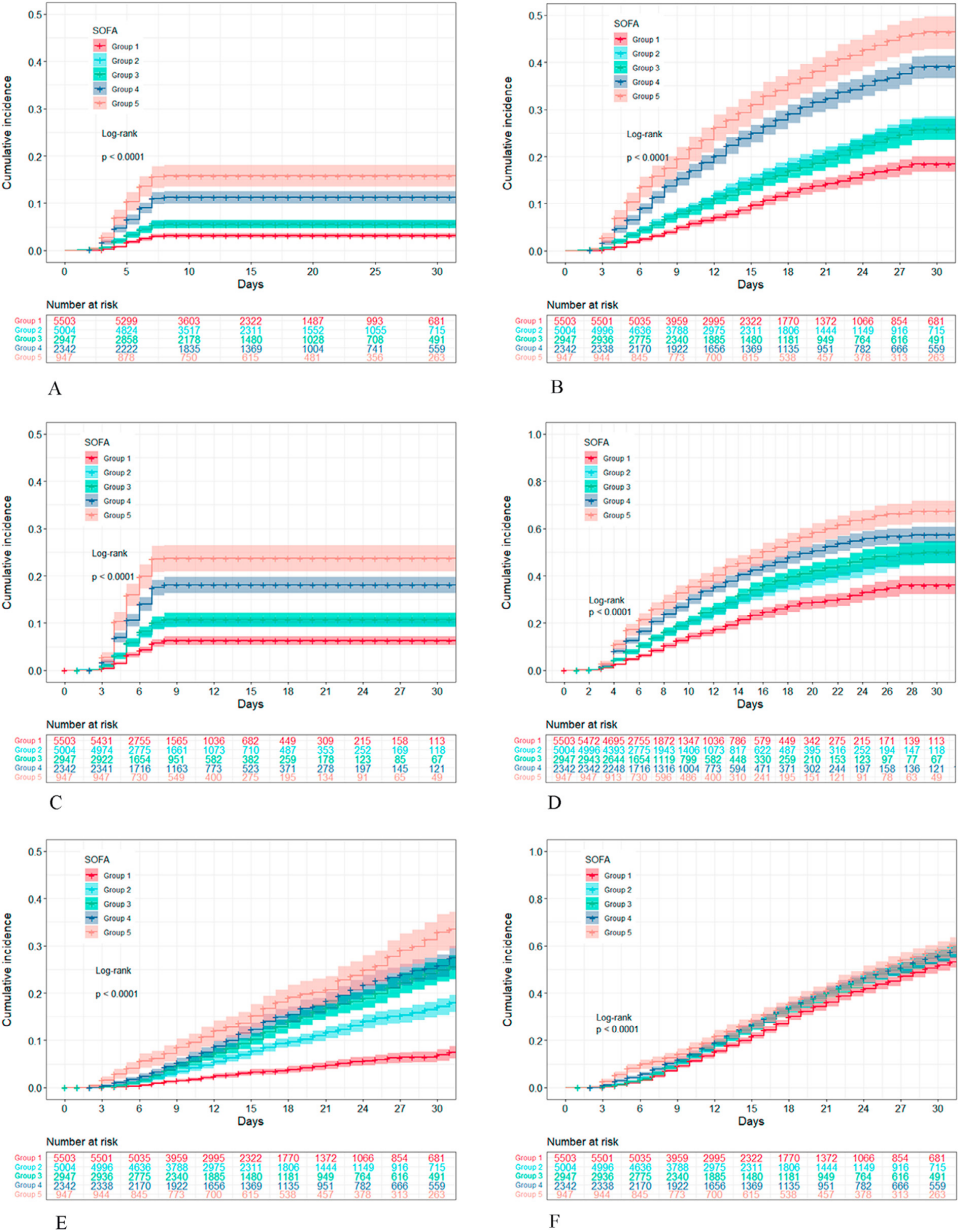
Cumulative incidence curves of patients with different trajectories of the SOFA score. (A) 7-day in-hospital mortality. (B) 28-day in-hospital mortality. (C) 7-day ICU mortality. (D) 28-day ICU mortality. (E) Incidence of septic shock. (F) Incidence of ARF. ARF: Acute respiratory failure; ICU: Intensive care unit; SOFA: Sequential Organ Failure Assessment.

The cumulative risk curve of adverse outcome according to the SOFA score of patients with sepsis is shown in Figure 3. For the 7- and 28-day survival outcomes, the risk curve of all trajectory groups exhibited a clear trend of gradual increase. In group 5, the high SOFA score group was associated with the highest cumulative risk. In group 1, the low SOFA score group was linked to the lowest cumulative risk. A statistically significant difference was found in the cumulative death risk for patients from all trajectory groups (*P*<0.001). For the outcome of septic shock, the risk curve of each trajectory group increased gradually from the third day after admission. The cumulative risks of groups 2–5 were gradually increased, while that of group 1 remained at a relatively low level. There were statistically significant differences in the cumulative risk of septic shock for patients in the trajectory groups (*P*<0.001). For the ARF outcome, the cumulative risk of all trajectory groups showed a similar increasing trend. Statistically significant differences were found in the cumulative risk of ARF among the patients classified in the trajectory groups (*P*<0.001).

### **Cox proportional hazards regression analysis of different clinical outcomes and SOFA trajectories**

In this study, the Cox proportional hazards regression model was used to evaluate the relationship between SOFA trajectory and adverse clinical outcomes in patients with sepsis. When fitting the model, group 1 was first used as the reference group to fit the univariate Cox model of the SOFA trajectory variables. Thereafter, the different covariables at baseline were adjusted in four different models. The results are shown in Table 3. In the univariate analysis, for the outcome of 7-day in-hospital mortality and using trajectory 1 as the reference group, the HR (95% CI) values for groups 2–5 were 1.893 (1.561–2.296), 1.803 (1.449–2.242), 3.786 (3.113–4.603), and 5.478 (4.387–6.841), respectively. The results showed that these groups were risk factors for 7-day in-hospital mortality (*P* < 0.001). After adjusting the multivariate model of different covariates, groups 2–5 demonstrated a similar survival status to that observed on day 7. The mortality were independently correlated with SOFA trajectory (*P* < 0.001). The findings suggested that patients with sepsis whose SOFA score gradually increased in the longitudinal change trajectory were at a higher risk of death within 7 days compared with those who did not exhibit such an increase.

**Table 3:** HR and 95% CI for predictors of trajectory group.

Variables	Non-adjusted		Model I		Model II		Model III	
	HR (95% CI)	*P*-value	HR (95% CI)	*P*-value	HR (95% CI)	*P*-value	HR (95% CI)	*P*-value
7-day in-hospital mortality								
Group 1	1.0 (Ref)		1.0 (Ref)		1.0 (Ref)		1.0 (Ref)	
Group 2	1.893 (1.561–2.296)	<0.001	1.792 (1.477–2.173)	<0.001	1.808 (1.486–2.198)	<0.001	1.787 (1.466–2.178)	<0.001
Group 3	1.803 (1.449–2.242)	<0.001	1.742 (1.400–2.168)	<0.001	1.781 (1.420–2.235)	<0.001	1.710 (1.352–2.161)	<0.001
Group 4	3.786 (3.113–4.603)	<0.001	3.778 (3.105–4.596)	<0.001	3.842 (3.105–4.753)	<0.001	3.490 (2.778–4.385)	<0.001
Group 5	5.478 (4.387–6.841)	<0.001	6.012 (4.804–7.523)	<0.001	6.668 (5.140–8.650)	<0.001	5.687 (4.253–7.605)	<0.001
28-day in-hospital mortality								
Group 1	1.0 (Ref)		1.0 (Ref)		1.0 (Ref)		1.0 (Ref)	
Group 2	1.605 (1.439–1.791)	<0.001	1.492 (1.337–1.665)	<0.001	1.438 (1.287–1.607)	<0.001	1.426 (1.275–1.596)	<0.001
Group 3	1.508 (1.333–1.707)	<0.001	1.432 (1.265–1.621)	<0.001	1.384 (1.216–1.575)	<0.001	1.356 (1.187–1.550)	<0.001
Group 4	2.696 (2.411–3.016)	<0.001	2.708 (2.421–3.030)	<0.001	2.519 (2.228–2.848)	<0.001	2.402 (2.105–2.739)	<0.001
Group 5	3.428 (3.009–3.905)	<0.001	3.901 (3.420–4.449)	<0.001	3.693 (3.170–4.303)	<0.001	3.411 (2.878–4.043)	<0.001
7-day ICU mortality								
Group 1	1.0 (Ref)		1.0 (Ref)		1.0 (Ref)		1.0 (Ref)	
Group 2	1.758 (1.482–2.085)	<0.001	1.657 (1.397–1.966)	<0.001	1.683 (1.416–1.999)	<0.001	1.574 (1.321–1.875)	<0.001
Group 3	1.825 (1.511–2.205	<0.001	1.744 (1.443–2.108)	<0.001	1.838 (1.512–2.233)	<0.001	1.632 (1.332–1.999)	<0.001
Group 4	3.309 (2.794–3.920)	<0.001	3.304 (2.788–3.915)	<0.001	3.320 (2.780–3.965)	<0.001	2.880 (2.362–3.511)	<0.001
Group 5	4.573 (3.780–5.534)	<0.001	5.087 (4.197–6.164)	<0.001	5.116 (4.166–6.283)	<0.001	4.316 (3.364–5.536)	<0.001
28-day ICU mortality								
Group 1	1.0 (Ref)		1.0 (Ref)		1.0 (Ref)		1.0 (Ref)	
Group 2	1.551 (1.395–1.725)	<0.001	1.441 (1.295–1.603)	<0.001	1.366 (1.226–1.522)	<0.001	1.285 (1.152–1.434)	<0.001
Group 3	1.620 (1.438–1.825)	<0.001	1.522 (1.350–1.715)	<0.001	1.437 (1.268–1.629)	<0.001	1.317 (1.159–1.498)	<0.001
Group 4	2.280 (2.047–2.541)	<0.001	2.250 (2.019–2.508)	<0.001	2.047 (1.816–2.307)	<0.001	1.807 (1.591–2.052)	<0.001
Group 5	2.823 (2.492–3.197)	<0.001	3.113 (2.746–3.528)	<0.001	2.921 (2.522–3.383)	<0.001	2.320 (1.972–2.729)	<0.001
Septic shock								
Group 1	1.0 (Ref)		1.0 (Ref)		1.0 (Ref)		1.0 (Ref)	
Group 2	2.353 (2.006–2.760)	<0.001	2.219 (1.891–2.604)	<0.001	1.998 (1.701–2.347)	<0.001	2.005 (1.705–2.359)	<0.001
Group 3	3.307 (2.813–3.888)	<0.001	3.123 (2.655–3.673)	<0.001	2.700 (2.286–3.188)	<0.001	2.633 (2.217–3.127)	<0.001
Group 4	3.752 (3.195–4.407)	<0.001	3.692 (3.143–4.338)	<0.001	3.154 (2.661–3.738)	<0.001	3.119 (2.603–3.737)	<0.001
Group 5	4.649 (3.884–5.563)	<0.001	5.066 (4.230–6.068)	<0.001	4.232 (3.462–5.174)	<0.001	4.305 (3.452–5.367)	<0.001
ARF								
Group 1	1.0 (Ref)		1.0 (Ref)		1.0 (Ref)		1.0 (Ref)	
Group 2	1.172 (1.101–1.248)	<0.001	1.142 (1.072–1.216)	<0.001	1.176 (1.103–1.253)	<0.001	1.173 (1.099–1.252)	<0.001
Group 3	1.304 (1.215–1.399)	<0.001	1.107 (1.031–1.188)	0.005	1.215 (1.128–1.308)	<0.001	1.202 (1.112–1.300)	<0.001
Group 4	1.081 (1.006–1.161)	0.034	1.152 (1.073–1.238)	<0.001	1.346 (1.245–1.455)	<0.001	1.304 (1.199–1.420)	<0.001
Group 5	1.122 (1.023–1.229)	0.014	1.261 (1.150–1.383)	<0.001	1.684 (1.515–1.872)	<0.001	1.600 (1.423–1.799)	<0.001

Models were derived from Cox proportional hazards regression models. Non-adjusted model adjust for: none.

Adjust model I adjust for: Age, Gender, Ethnicity, Insurance, Marital status.

Adjust model II adjust for: Model I plus RRT, Vent, Drug, CCI, Glucose, HeartRate, SBP, DBP, MBP, Respir, SpO_2_, Temper, PaCO_2_, PaO_2_.

Adjust model III adjust for: Model II plus WBC, Bicarbsonate, Chloride, Hematocrit, BCR, INR, RDW, Anion gap, Hemoglobin, Platelet.

ARF: Acute respiratory failure; BCR: Blood urea nitrogen and creatinine ratio; CCI: Charlson Comorbidity Index; CI: Confidence interval; DBP: Diastolic blood pressure; HR: Hazard ratio; ICU: Intensive care unit; INR: International normalized ratio; MBP: Mean arterial pressure; PaCO_2_: Artial pressure of carbon dioxide; SpO_2_: Oxygen saturation; Ref: Reference; RDW: Red cell distribution width; RRT: Renal replacement therapy; SBP: Systolic blood pressure; WBC: White blood cell count.

The in-hospital mortality within 28 days was also analyzed. Similarly, univariate and multivariate Cox regression analyses using trajectory group 1 as a reference also showed that groups 2–5 continued to be independently correlated with 28-day mortality (*P* < 0.001). Finally, univariate Cox proportional hazards regression analysis was performed to examine the relationship between the occurrence of septic shock and the longitudinal trajectory of the SOFA score. The analysis showed that, using group 1 as the reference group, the HR (95% CI) values for groups 2–5 were 2.353 (2.006–2.760), 3.307 (2.813–3.888), 3.752 (3.195–4.407), and 4.649 (3.884–5.563), respectively. These results showed that these groups were independent risk factors. Adjustment for different covariates demonstrated that groups 2–5 continued to be independently related to the occurrence of septic shock and ARF (*P* < 0.001). Patients in group 5 were at the highest risk for septic shock and ARF (*P* < 0.001).

### **Subgroup analyses**

**Figure 4:**
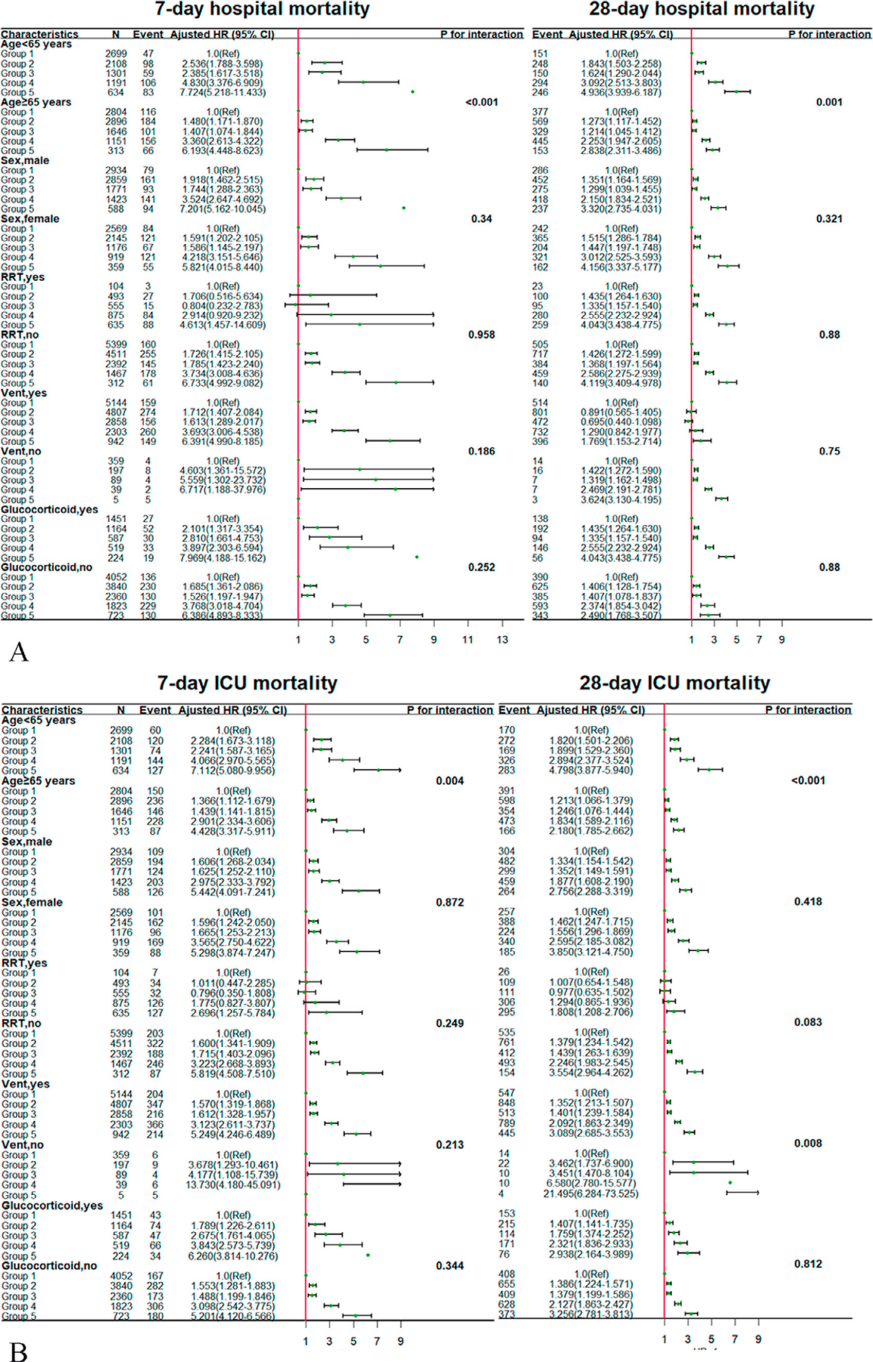

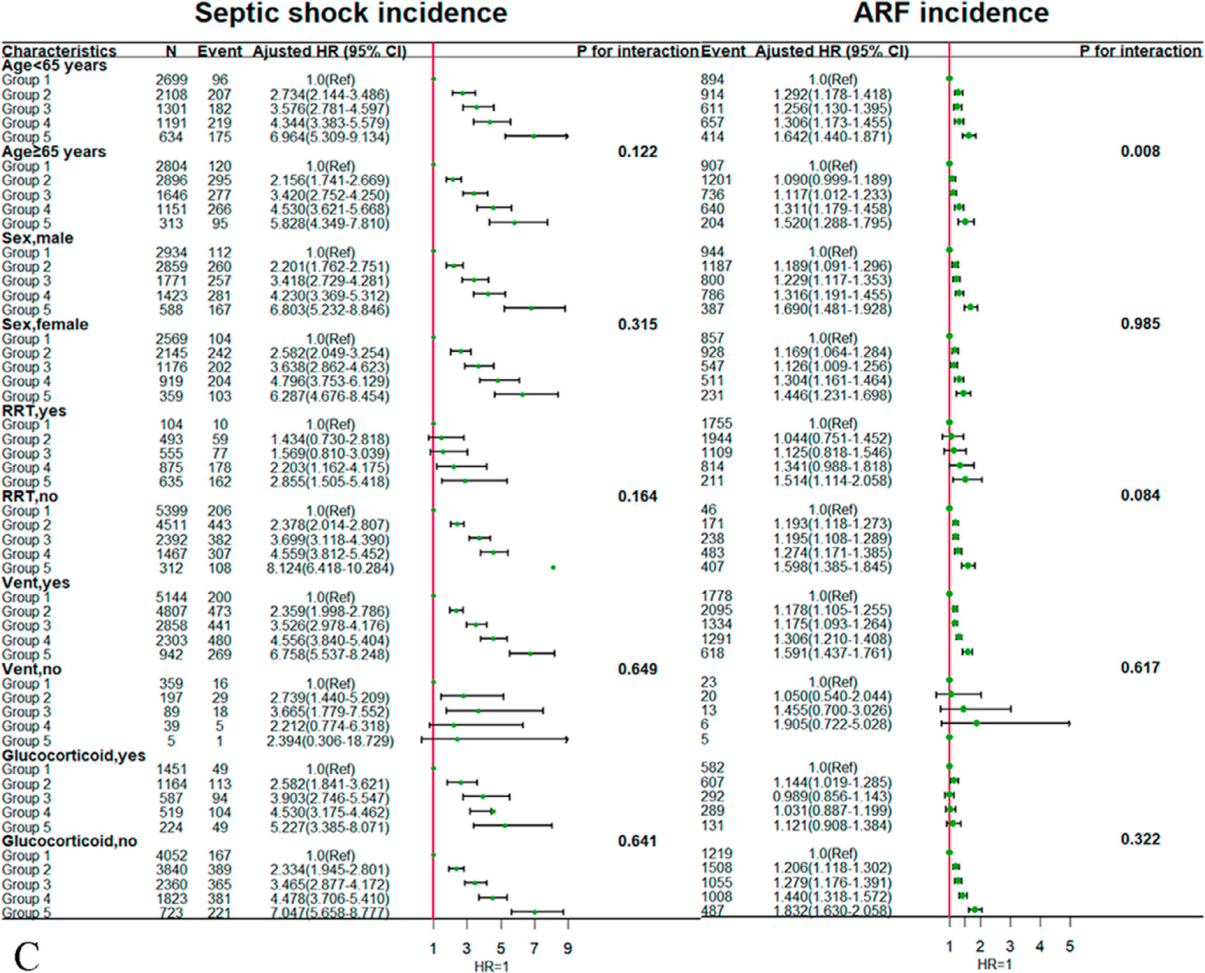
Subgroup analysis for associations between SOFA score trajectories and adverse outcomes according to baseline characteristics. (A) In-hospital mortality. (B) ICU mortality. (C) Incidence of septic shock and ARF. ARF: Acute respiratory failure; CI: Confidence interval; HR: Hazard ratio; ICU: Intensive care unit; RRT: Renal replacement therapy;Ref: Reference; SOFA: Sequential Organ Failure Assessment.

We selected the following as research variables for the stratified subgroup analysis: age, sex, use of RRT, use of mechanical ventilation, and administration of glucocorticoid therapy. In the age-stratified analysis, patients with sepsis aged <65 years were at a significantly higher risk of death according to the SOFA trajectory for the survival status in the hospital and ICU compared with those aged ≥65 years (*P* < 0.05). For the 28-day mortality in the ICU, patients who did not receive mechanical ventilation were at a significantly higher risk of death according to the SOFA trajectory compared with those who received mechanical ventilation. For the outcome of ARF, patients with sepsis aged <65 years were at a significantly higher risk of death according to the SOFA trajectory compared with those aged ≥65 years (*P* = 0.008). The results of the subgroup analysis are shown in detail in Figure 4.

## Discussion

Sepsis remains the leading cause of death in critically ill patients.^[3]^ Early observation and monitoring of changes in the patient's condition and active use of effective treatments are of great importance to the prognosis. This type of practice can assist in reducing mortality.^[24]^ The SOFA score, proposed by the European Society of Critical Care Medicine,^[25]^ is an objective system utilized to evaluate the severity of organ dysfunction or failure in patients with sepsis. This score is one of the necessary items for the diagnosis of patients with sepsis.^[26]^ Thus, dynamic monitoring of the change trajectory of the SOFA score in patients with sepsis is crucial.

In this study, we grouped patients with sepsis into trajectory groups according to their SOFA scores based on the univariate GBTM. GBTM was used to monitor changes in the SOFA score and determine the disease development trajectory in patients with sepsis. According to the fitting results of the model, we divided the SOFA scores of patients with sepsis into five trajectory groups. In the Cox proportional hazards regression analysis, following analysis of adjusted and unadjusted covariates, a more uniform pattern was evident. Trajectory groups with higher SOFA scores were associated with an increased risk for various adverse outcomes. The SOFA score consists of six parts: breathing, coagulation, liver, circulation, nerves, and kidneys. The score for each of these parts ranges from 0 (no organ dysfunction) to 4 (severe organ dysfunction) points. The scores for each part are added, and the final total score (0–24) is used to reflect the function of multiple organs throughout the body.[3], [27]

Initially, the SOFA score of group 1 ranged 0–4 points and showed a downward trend after 20 h. These results indicated limited organ damage and gradual improvement. Higher SOFA scores reflected greater initial damage to organ function in patients.^[25]^ Patients in groups 2–5 showed an upward trend in the early stage, reflecting the increase in organ damage and the number of damaged organs, indicating poor prognosis. We observed that the SOFA score of group 3 exhibited a relatively obvious downward trend; nevertheless, it remained higher than that observed for group 2. Cox proportional hazards regression analysis revealed that patients in group 3 were at lower risk of in-hospital death than those in group 2. However, the risk of death and septic shock in the ICU and the risk of ARF in this group remained higher than those noted in group 2.

The decrease in SOFA score reflected the effectiveness of early treatment in patients with sepsis. However, following a decrease to a certain level, it showed a trend toward stabilization. Possible reasons for this observation include inadequate control of the infection, disease recurrence, or damage to an organ that could not be promptly corrected.^[28]^ Therefore, although patients in group 3 exhibited a significant downward trend, their prognosis remained relatively poor. A randomized controlled trial showed that changes in the SOFA score could reliably reflect the mortality of patients with sepsis.^[29]^ Another prospective cohort study showed that the SOFA score in the first few days of ICU admission was a good indicator of prognosis, while an increase in this score within the first 48 h of ICU admission could effectively predict death.^[30]^

The present study was based on a GBTM analysis of the longitudinal trajectory changes in the SOFA scores of patients with sepsis. The results showed that groups 2–5 were independent risk factors for the occurrence of various adverse outcomes; specifically, higher SOFA scores were associated with poor prognosis in patients with sepsis. The severity of disease in patients with sepsis and related influencing factors can be identified by exploring the change trajectory in the SOFA score of patients with sepsis within 72 h after admission, analyzing the possible trajectory subgroup types, and determining the prognosis of different trajectory subgroups. This provides a basis and guidance to clinicians for the diagnosis and treatment of patients with sepsis.

We further performed a subgroup analysis based on covariates, such as age, sex, RRT, mechanical ventilation, and administration of glucocorticoids. Age remained a predictor of mortality and ARF incidence. The mortality of patients aged <65 years in each group of SOFA score was significantly higher than that of patients aged ≥65 years. Previous studies suggested that the risk of sepsis had a bimodal distribution: higher in infants; lower in young individuals; and higher in those aged >60 years.^[31]^ Our findings are not consistent with that observation. Older patients have more complications due to diabetes, hypertension,^[32]^ aging of the immune^[33]^ and neuroendocrine systems,^[34]^ and stress. In addition, the compensatory ability of older patients to external stimuli (e.g., trauma^[35]^ and inflammation^[36]^) is weaker than that of young individuals. For example, in the case of early sepsis in patients with renal dysfunction due to microcirculation or severe inflammation, younger patients demonstrated greater compensatory capacity, and the urine output may have not been markedly reduced.[20], [37], [38] However, due to their weak compensatory ability, the level of urine output in elderly patients was significantly decreased. In such cases, the scores of kidney function in older patients were higher than those in recorded in younger patients; however, patients in both groups suffered kidney damage.

The use of mechanical ventilation as a predictor of 28-day mortality^[39]^ was also investigated. In all trajectory groups, the mortality rate among patients who did not receive mechanical ventilation was significantly higher than that noted in patients who received mechanical ventilation. Clinically, patients with sepsis are prone to complications with ARDS or severe lung inflammation.^[40]^ Previous studies have revealed that timely treatment with a ventilator can relieve tissue hypoxia, lessen lung work, and reduce the occurrence of complications. This approach prolongs the life of patients and improves their survival rate in later stages.^[41]^

## Strengths and Limitations

This study is aiming to determine the relationship between the SOFA score trajectory and poor prognosis in patients with sepsis, which involves a GBTM analysis of data obtained from the MIMIC-IV database. We determined the SOFA score trajectory, admission characteristics, and results of five different groups of patients with sepsis. This study used GBTM to determine the different trajectory classifications of the SOFA scores of ICU patients with sepsis, cluster the populations to identify the trajectory populations at high risk of adverse outcomes, and identify the characteristic trajectories of poor SOFA development trends. The present evidence provides a reference basis for clinicians to pay attention to nursing care and implement early intervention measures for key populations in clinical practice.

Our research also had some limitations. First, the present study was an observational investigation that only shows statistical associations with mortality and adverse outcomes.

Second, because this study only included patients in the USA, the relevance of extrapolating these results to ICU patients in other countries is uncertain.Finally, this was a retrospective observational study. Although the quality of the MIMIC-IV database is very high, data loss and input errors exist. Therefore, further studies are warranted to verify these results.

## Conclusions

We used GBTM to longitudinally cluster SOFA score data of patients with sepsis. We found that higher SOFA scores were linked to greater risk of poor prognosis. The present findings may identify potential risks for various health outcomes in different patient groups, as well as guide future interventions with research and clinical application value.

## Ethics Statement

The MIMIC-IV database has been approved by the Massachusetts Institute of Technology and Beth Israel Deaconess Medical Center, and consent for the collection of the original data was provided by the patients (https://physionet.org/content/mimiciv/view-license/1.0/). Therefore, ethical approval and the need for informed consent were waived for studies using data from this database.

## Data Availability

The MIMIC-IV data are available at https ://mimic-iv.mit.edu/. Nevertheless, the validation set generated for this study is not readily available because the ethics committee does not allow the release of the data. The dataset is available from the corresponding author upon reasonable request.

## Funding

This research did not receive any specific grant from funding agencies in the public, commercial, or not-for-profit sectors.

## Conflicts of Interest

The authors declare that they have no known competing financial interests or personal relationships that could have appeared to influence the work reported in this paper.

## Appendix Supplementary materials

Image, application 1
