# Supplementary material for: Analysis of the correlation between the longitudinal trajectory of SOFA scores and prognosis in patients with sepsis at 72 hour after admission based on group trajectory modeling
Source: J Intensive Med. 2021 Dec 21;2(1):39–49. doi: 10.1016/j.jointm.2021.11.001 (PMC9923968; doi:10.1016/j.jointm.2021.11.001)
Supplement: Supplementary file 1 [file mmc1.docx]

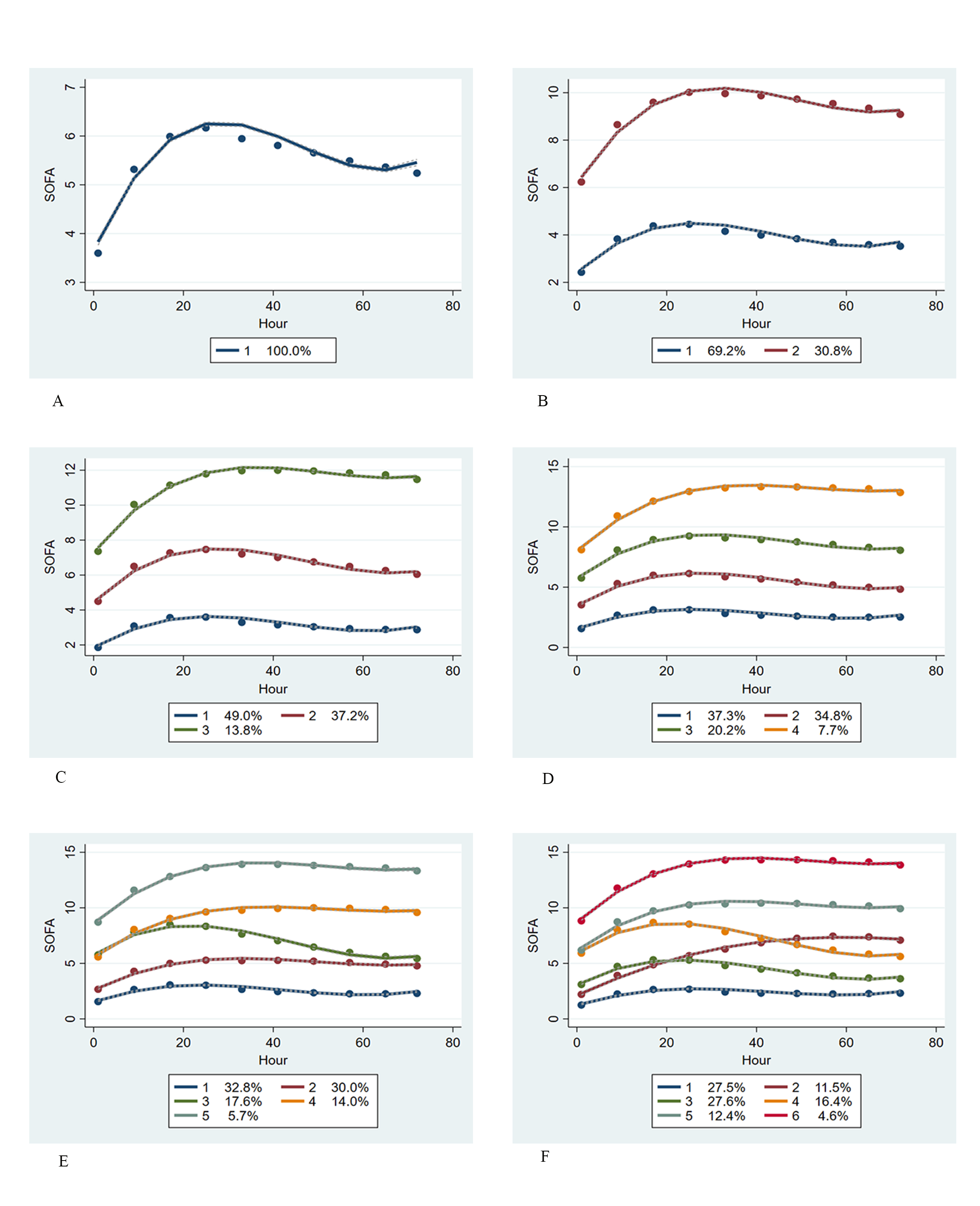


**Figure S1.** Trajectory group classification. The five identified trajectory groups of SOFA score represented by the columns.

**Supplementary materials**

**Table S1.**Parameter estimation of SOFA score fitting CNORM trajectory curve.

| Trajectory groups | Trajectory trends | Parameter | Estimates | Standard error | t | P |
| --- | --- | --- | --- | --- | --- | --- |
| 1 | Class 1 | Intercept | 1.231 | 0.032 | 38.550 | <0.001 |
|  |  | Liner | 0.172 | 0.003 | 50.980 | <0.001 |
|  |  | Quadratic | -0.005 | 0.000 | -45.590 | <0.001 |
|  |  | Cubic | 0.000 | 0.000 | 40.230 | <0.001 |
| 2 | Class 2 | Intercept | 2.496 | 0.042 | 60.010 | <0.001 |
|  |  | Liner | 0.215 | 0.004 | 58.380 | <0.001 |
|  |  | Quadratic | -0.005 | 0.000 | -37.310 | <0.001 |
|  |  | Cubic | 0.000 | 0.000 | 27.300 | <0.001 |
| 3 | Class 3 | Intercept | 5.686 | 0.053 | 107.975 | <0.001 |
|  |  | Liner | 0.283 | 0.005 | 58.067 | <0.001 |
|  |  | Quadratic | -0.009 | 0.000 | -52.081 | <0.001 |
|  |  | Cubic | 0.000 | 0.000 | 43.043 | <0.001 |
| 4 | Class 4 | Intercept | 5.504 | 0.054 | 102.180 | <0.001 |
|  |  | Liner | 0.294 | 0.005 | 55.448 | <0.001 |
|  |  | Quadratic | -0.006 | 0.000 | -34.303 | <0.001 |
|  |  | Cubic | 0.000 | 0.000 | 24.347 | <0.001 |
| 5 | Class 5 | Intercept | 8.543 | 0.069 | 123.095 | <0.001 |
|  |  | Liner | 0.368 | 0.008 | 46.079 | <0.001 |
|  |  | Quadratic | -0.008 | 0.000 | -29.832 | <0.001 |
|  |  | Cubic | 0.000 | 0.000 | 21.416 | <0.001 |
